# Aspects of social support and disclosure in the context of institutional abuse – long-term impact on mental health

**DOI:** 10.1186/s40359-015-0077-0

**Published:** 2015-06-19

**Authors:** Brigitte Lueger-Schuster, Asisa Butollo, Yvonne Moy, Reinhold Jagsch, Tobias Glück, Viktoria Kantor, Matthias Knefel, Dina Weindl

**Affiliations:** Faculty of Psychology, University of Vienna, Liebiggasse 5, 1010 Vienna, Austria

**Keywords:** Institutional abuse, Disclosure, Social support, Hostility, Mental health

## Abstract

**Background:**

The psychological sequelae of institutionalized abuse and its long-term consequences has not been systematically documented in existing literature in regarding social support once disclosure has been made. Reporting abuse is crucial, in particular for adult victims of childhood IA within the Catholic Church. Nevertheless, there is ongoing controversy about the benefits of disclosure. Our study examines the interaction of disclosure and subsequent social support in relation to mental health. We look into the times of disclosure, the behaviour during the disclosure to a commission as adults, different level of perceived social support, and the effect on mental health.

**Methods:**

The data were collected in a sample of financially compensated adult survivors who experienced institutionalized abuse during their childhood, using instruments to measure perceived social support, reaction to disclosure, PTSD, and further symptoms.

**Results:**

High levels of perceived social support after early disclosure result in a higher level of mental health and contribute to less emotionally reactive behaviour during disclosure of past institutionalized abuse. Highly perceived levels of social support seem to play a crucial role in mental health, but this inference may be weakened by a possible interference of a lasting competence in looking for social support versus social influences.

**Conclusion:**

Future research should thus disentangle perceived social support into the competence of looking for social support versus socially influenced factors to provide more clarity about the positive association of perceived social support and mental health.

## Background

For many years, the extent of institutionalized abuse during childhood perpetrated by representatives of the Catholic Church was unknown and not discussed publicly. However, in recent years, many countries and national Catholic Churches started victim compensation programs for the survivors of institutionalized abuse (Flanagan-Howard et al. 2009). In Austria, an “Independent Victim Protection Commission and Advocacy” was established in April 2010. Survivors were given the opportunity to contact the commission and report their experiences. When contacting this commission the survivors were given addresses from mental health experts. These mental health experts explored the scope of the abuse, gave crisis support, and produced a written report, which functioned as a basis for the amount of financial compensation as well as the financial amount dedicated for treatment hours. The core data from these reports were evaluated (e. g. was the person in that time in this institution? Was the perpetrator in that time in the institution?). The reports were than discussed by the members of the commission to take the decision about the amount of money and treatment hours for each evaluated case. The commission compensated 1700 survivors with a sum of 16.8 Mio € within the last five years, covering compensation and 45000 treatment hours. It is not possible to assume how many people were affected by institutional abuse by representatives of the Austrian Catholic Church, activists proclaim that the estimated number of unknown cases is about tenfold higher than the group who was already compensated. The money was given uniquely form the Austrian Catholic Church (www.opferschutz.at^2999^). The majority of these cases happened in the period from 1950 to 1970. Some of these survivors spoke for the first time about their abuse and most were severely affected by these experiences (Lueger-Schuster et al. 2014). This study investigated adult survivors who made disclosures to the commission after they had received financial compensation.

Child abuse includes many acts of all types of violence by an adult over a longer period of time (Lueger-Schuster et al. 2014) that often is related with mental health problems (Putnam et al. 2013). Childhood institutionalized abuse takes place in settings that do not need to be residential in the first place, where the child is controlled in most aspects by an institution or a single person. It entails the inappropriate use of power and authority, including the potential to harm a child’s well-being and development and creates the feeling of betrayal, stigmatization and powerlessness (Wolfe et al. 2003).

Multiple studies report negative effects of childhood abuse on mental health in adult survivors, such as PTSD, major depression, anxiety disorders, eating disorders and suicide attempts for example (Chen et al. 2010). However, the psychological impact of clerical institutionalized abuse has scarcely been investigated, but the effects seem to be highly adverse (Flanagan-Howard et al. 2009; Lueger-Schuster et al. 2014; Wolfe et al. 2003).

Child abuse coerce poorer mental health outcomes in adulthood, but some survivors experience lower impairment or even stay healthy. This applies also for survivors of institutionalized abuse (Carr et al. 2010). Several factors moderate the impairment, among those disclosure, social support, and social affective reactions that are considered a mental state that refers to both the self and others. Izard (Izard 1971) saw anger as one of the social affective reactions within the hostility triad, involving hostile tendencies towards other persons. Especially anger phenomena are frequent in the context of traumatic stress (Olatunji et al. 2010). Anger and aggression after the experience of sexual abuse have also been frequently reported (Briere & Elliott 2003; Hillberg et al. 2011). This may further be a function of the betrayal experienced after the abuse occurred (Finkelhor & Browne 1985). Specifically in individuals who suffered of institutionalized abuse during their childhood the betrayal aspect might be held responsible for a variety of outcomes, *e.g.* interpersonal problems (Smith & Freyd 2014), a higher risk to meet criteria for personality disorders (Carr et al. 2010), and problems with self regulation (Ehring & Quack 2010). To our knowledge aspects of disclosure and social support in relation with posttraumatic stress symptoms and anger phenomena, *e.g.* hostility have not been investigated in a male dominated sample of adult survivors of institutionalized abuse so far.

### Social support

Social support for individuals exposed to traumatic stress is apparently an important factor when coping with traumatic stress (Brewin et al. 2000). Generally, social support is acknowledged as a factor in relation to its positive effects on disorders and mental health (Kaniasty & Norris 2008). Social support indicates a low to medium correlation with PTSD (Brewin et al. 2000). Furthermore, the health promoting impacts of social support on the consequences of child sexual abuse are evident (Stevens et al. 2013).

Social support influences health by two models: the main effect model and the stress buffering model (Cohen & Syme 1985). The main effect model follows the idea that social support improves a person’s health through guidance on healthy behaviour, by improving self-esteem, and by increasing the sense of belonging, whereas the stress buffering model of social support prevents from damaging responses, and thus health improves. Results from a study with adult women suffering from multiple forms of child abuse and neglect support both direct and mediational effects of social resources on PTSD and depression in adulthood (Vranceanu et al. 2007). Moreover, the definitions of social support are heterogeneous and several terms coexist in parallel (Guay et al. 2006). Perceived support reflects the subjective judgments of the support given, and is consistently linked with fewer PTSD symptoms (Brewin et al. 2000). Survivors of sexual abuse with a higher level of perceived social support experienced lower levels of insomnia, nightmares and nightmare distress (Steine et al. 2012). In a study with older adults (aged from 57 to 85 years) a perceived lack of social support was associated with lower levels of physical health (Cornwell & Waite 2009). There is a rather substantial support that perceived social support buffers the rate and severity of psychopathology (e. g. depression, anxiety, psychological distress), resulting from traumatic stress (Cohen & Wills 1985; Brewin et al. 2000). However, the relation between social support and chronic PTSD is less well understood, than the role of social support in the onset of PTSD. Low social support and the development of PTSD has been found to be associated in cross-sectional studies in samples of victims of violent crimes (Andrews et al. 2003), and in women with sexual and nonsexual assault (Zoellner et al. 1999).

However, social integration and perceiving social support are not independent of knowledge shared about the assault. Apart from the possibility of reaching helpful aid, the process of revealing the abuse to someone is also considered to have an emotionally adverse impact (Smith & Freyd 2014). To our knowledge, so far there is no study on the role of social support in survivors of institutionalized abuse.

### Disclosure

Empirical studies suggest that among survivors only few children tell anyone about sexual abuse. Despite the high prevalence of abuse, child victims often fail or delay to tell others about their abuse (Ullman SE. Social reactions to child sexual abuse disclosures: a critical review. Journal of Child Sexual Abuse 2002). Adult males are less likely to disclose their childhood sexual abuse experience compared to female victims (O’Leary & Barber 2008; Lamb & Edgar-Smith 1994). The rates of disclosing child physical abuse, child sexual abuse, and emotional abuse show that 23 % to 34 % of the victims fail to ever disclose their adverse experience, depending on the type of abuse (Bottoms et al. 2014). Disclosing abuse is often difficult, resulting in possible reactions of disbelief, blame or challenges to relationships (Ullman & Filipas 2001). For emotional and physical abuse a close victim-perpetrator-relationship explains the delay of disclosure or keeping the adverse experience silence (Foynes et al. 2009). Depending on the care of an abusive caregiver is a pathway into a dilemma: disclosing might cut off the caring relation, non-disclosing would prolong the abusive situation (Foynes et al. 2009). Reasons for disclosure and non-disclosure, *e.g.* severity of trauma, being injured by the abuser (O’Leary et al. 2010) are believed to influence the timing of disclosure. Several different time frames to distinguish between early and late disclosure have been considered; however, no theoretical explanations have been provided for these (Ruggiero et al. 2004).

Although several studies have investigated the impact of disclosure on mental health, their results are inconsistent (Müller et al. 2008). Esterling, L’Abate, Murray, and Pennebaker (Esterling et al. 1999) discovered long-term improvements on mental health. Contradicting results were found by O’Leary *et al.* (O’Leary et al. 2010); early disclosure was associated with a greater number of symptoms than late disclosure. No correlation at all between disclosure and PTSD symptoms was found by Glover *et al.* (Glover et al. 2010). For males, years until disclosure, overall response to the disclosure, the use of physical force by the abuser, number of childhood adversity, and conformity of masculine norms were predictive for mental distress (Easton 2014). Further research would clarify the effects of the timing of disclosure.

Moreover, aspects of the reaction to the disclosure may impact the survivors’ ability to adjust. The reactions during disclosure may be reciprocal with the reaction to disclosure, *e.g.* a distressed person may be more emotional when making a disclosure and might receive more of an emotional reaction from the person to whom he or she is disclosing the abuse (Ullman SE. Social reactions to child sexual abuse disclosures: a critical review. Journal of Child Sexual Abuse 2003). Dysfunctional disclosure tendencies, *e.g.* reluctance to disclose, a strong urge to talk about it, and bodily as well as emotional reactions during the disclosure are related to poorer mental health (Pielmaier & Maercker A. Psychological adaptation to life-threatening injury in dyads: The role of dysfunctional disclosure of trauma. European journal of psychotraumatology 2011).

### Hostility

Several studies show the relation of feeling helpless and aggression respectively hostility (Jakupcak & Tull 2005; Czaja & Gierowski 1998). Anger and aggression have been frequently reported after the experience of sexual abuse (Briere & Elliott 2003; Hillberg et al. 2011). Especially in the case of institutionalized abuse this may further be a function of the betrayal and injustice experienced after the abuse occurred (Finkelhor & Browne 1985). Maercker and Horn (Maercker & Horn 2013) placed anger, along with shame and guilt, in their socio-interpersonal model as an important factor as a social affective response at the individual level that influences posttraumatic outcome. In meta-analytic studies it was shown that anger and aggression are strongly related to PTSD and the maintenance of symptoms with the effect of anger becoming stronger over time, adding significantly to symptom distress (Orth & Wieland 2006). Anger rumination and hostile anticipation in the form of revenge planning is potentially important in explaining anger and aggression in this sample, because when they were children they could not act out the aggression and anger caused by their perpetrators. Aspects specifically anger and hostility have not yet been investigated thoroughly in trauma survivors.

To our knowledge the relation between disclosure, perceived social support, and hostility is still unclear.

### Purpose

The purpose of the study is to examine the interaction of disclosure and perceived social support in relation to mental health. In detail, we investigate the time in which the disclosure was made (before versus after the age of 18, using the age of 18 as indicator for the first disclosure when being an adult) in combination with the amount of perceived social support at the time of the first disclosure after past institutionalized abuse and relate these factors with the level of mental health symptoms in nine dimensions. These dimensions are: posttraumatic stress symptoms, the reactions during the current disclosure when the individuals addressed themselves to the commission. We expect higher emotional disclosure and a higher level of reluctance to talk in connection with a higher level of verifiable symptoms in the recent disclosing group compared to those who broke their silence during childhood and have perceived a higher degree of social support. Further, we look for predictors for the severity of hostility as one of the dominant social affects for the level of symptoms.

## Methods

### Procedure and participants

Ethical clearance to the study protocol was given by the University of Vienna Ethics Committee. The study was also listed in the WHO approved German Clinical Trials Register (DRKS-ID: DRKS00003222). Written informed consent prior to receiving the questionnaires was obtained by all participants.

As a result of numerous disclosures by survivors of child abuse committed by representatives of the Catholic Church, the cardinal of Vienna implemented an independent victim protection commission. Survivors were given the possibility of disclosing their experiences of violence and depending on their experience, voluntary financial compensation and psychotherapeutic help were offered (Lueger-Schuster et al. 2014).

795 survivors who were already compensated by the commission were invited to participate in our study, and 448 consented to the analyses of their documents containing all the information derived from interviews with clinical psychologists and psychotherapists about their adverse experiences caused by representatives of the Catholic Church. The sample size was rather satisfying at the time, when data collection took place. Data were collected from August 2011 to May 2012. Of these 448 individuals, 163 (36.4 %) completed a set of clinical questionnaires including information about the time of the first disclosure. 125 (76.7 %) were males and 38 (23.3 %) females; the average age of the participants was 55.73 (SD = 9.34, range = 26–80). Most participants are married or cohabiting n = 98 (60.5 %), while n = 64 (39.5 %) have another relationship status. Most of the participants graduated from an apprenticeship or vocational school (n = 75, 46.6 %), while n = 60 (37.3 %) attended high school or university, and n = 26 (16.1 %) have no compulsory schooling. In comparison to the survivors not participating in the questionnaire survey, there were no significant differences concerning age, gender, marital status or education (all p > .05). The majority of adult survivors (83.3 %) experienced emotional abuse. Rates of sexual (68.8 %) and physical abuse (68.3 %) were almost equally high. The prevalence of PTSD was 48.6 % and 84.9 % showed clinically relevant symptoms (Lueger-Schuster et al. 2014).

### Measures

#### Social support

The Recalled Perceived Social Support Questionnaire (RPSSQ) was developed by a part of the research team to measure perceived social support after institutional abuse on three time levels, *i.e.* before the abuse (6 items), right after the abuse (10 items) and today (6 items). The first item of the instrument is “There were people in whom I could trust” for time level 1 (before) and 2 (after) being modified in “There are people in whom I can trust” for time level 3 (today). Specifically, for this study we asked for perceived social support in the time immediately after the onset of abuse. The 10 items measure on a five-point Likert scale (0 = “does not apply to at all” to 4 = “totally applies to”) perception of emotional support, practical support and social integration after the abuse. The score ranges from 0–40 with higher scores indicating a higher level of perceived social support. The construction of the questionnaire was based on questionnaires of Schulz and Schwarzer (Schulz & Schwarzer 2003), and Sommer and Fydrich (Sommer & Fydrich 1989). We obtained a Cronbach’s α = .79 in our sample.

#### Intensions and emotions during disclosure

The Disclosure of Loss Experience Scale; DLE; (Müller et al. 2011) is a 12-item version of the Disclosure of Trauma Scale (Mueller et al. 2009). It measures intentions to talk and emotions during disclosure on a six-point Likert scale (0 = “I agree not at all” to 5 = “I agree completely”). The DLE includes three subscales (“urge to talk”, “emotional reactions” and “reluctance to talk”) with satisfactory reliability (Cronbach’s α = .77 for the total score and ranged from α = .70 to α = .89 for the three subscales).

#### PTSD symptoms

The Posttraumatic Stress Disorder Checklist – Civilian Version; PCL-C; (Steine et al. 2012) examines 17 symptoms of PTSD based on the DSM-IV with good psychometric properties to reliably detect PTSD. Participants rate how often they have experienced symptoms in the past four weeks on a five-point Likert scale (0 = “*none*” to 4 = “*very*”). Cluster B (Re-Experiencing) consists of 5 items (*e.g.* flashbacks, nightmares), cluster C (Avoidance) of 7 items (*e.g.* avoidance of activities, emotional numbing), and Cluster D (Hyperarousal) of 5 items (*e.g.* being over-alert, being irritable and nervous). The total score ranges from 0–68. For this study, the German translation of the PCL-C (Teegen 1997) was used. Cronbach’s α ranged from α = .84 to α = .88 for the three symptom clusters with a Cronbach’s α = .93 for the total score).

#### Comorbid symptoms and hostility

The Brief Symptom Inventory; BSI; (Derogatis & Melisaratos 1983) is a valid and reliable self-report measure of clinically relevant psychological symptoms. Participants rate 53 items relating to their symptom distress for the past seven days on a five-point Likert scale (0 = “*not at all*” to 4 = “*extremely*”). For this study the German translation was used (Franke & Derogatis 2000). The reliability measures ranged from Cronbach’s α = .71 to α = .87 for the nine subscales with a Cronbach’s α = .97 for the total score. Within the BSI the hostility scale consist of 5 items, which are “Feeling easily annoyed or irritated”, “Temper outbursts that you could not control”, “Having urges to beat, injure or harm someone”,” Having urges to break or smash something”, “Getting into frequent arguments”. The reliability measure for the hostility scale is Cronbach’s α = .75.

### Data analysis

All statistical analyses were conducted using SPSS 20.0 for Windows. Categorical data were investigated with Chi-squared tests. Three MANOVAs were computed for each of the three outcome instruments with the subscales as dependent variables and time of disclosure (childhood vs. adulthood, cut-off = 18 years) as the independent variable, perceived social support was used as covariate. Pillai’s trace was used as test parameter, as effect size measure partial Eta-squares were calculated (low: Eta^2^ < .01, medium: Eta^2^ <. 06, high: Eta^2^ < .14). After this, we computed ANOVAs to compare the means of the four groups, regarding the mental health outcomes. Additionally a binary-logistic regression was carried out to look for predictors for the severity of hostility (clinically relevant defined as T-score of 63 and above) which is characteristic for a population that experienced IA. The alpha was set at a *p* < .05. As two of the samples were small in size (n < 30), *p*s < .10 were interpreted as a tendency to significance.

## Results

At the time of exposure to IA the participants were 9.81 years of age (SD = 3.06; Min 2, Max 16), early disclosure took place when they were between 4.5 and 18 years old (M = 10.99, SD = 3.25). The average time of the delay of disclosure was 18.8 years (n = 153, SD = 18.19). From n = 162 participants, disclosure was made to mothers (29.9 %), other family members (13.4 %), friends and partners (29.1 %), and 36.9 % reported the abusive experiences to authorities, e. g. teachers. Table 1 shows the sociodemographic characteristics of the study population.

**Table 1:** **Sample characteristics of study population**

Gender	Male	Female
N (%)	125 (76.7 %)	38 (23.3 %)
Age at the time of testing		
(in years)	mean (SD)	range
	55.73 (9.34)	26–80
Marital Status^a^		
N (%)	married/cohabited	other
	98 (60.5 %)	64 (39.5 %)
Highest level of formal education^b^		
N (%) None/compulsory	apprenticeship/vocational school	high school/university
26 (16.1 %)	75 (46.6 %)	60 (37.3 %)

*Note*. ^a^N = 162. ^b^N = 161

In terms of the variables on the status of mental health at the time of the survey, the multivariate analysis showed a significant result for perceived social support (*F*_(10, 145)_ = 2.087, *p* = .029, Eta^2^ = .123), but not for timing of disclosure (*F*_(10, 145)_ = 0.656, *p* = .763). In the second multivariate analysis with the three DLE subscales as dependent variables perceived social support yielded a significant result (*F*_(3, 152)_ = 3.243, *p* = .024, Eta^2^ = .058), while timing of disclosure (*F*_(3, 152)_ = 0.430, *p* = .732) did not. In the third multivariate analysis with the PCL-C scales as dependent variables perceived social support yielded a trend to significance (*F*_(3, 152)_ = 2.460, *p* = .065, Eta^2^ = .046), but a non-significant result for timing of disclosure (*F*_(3, 152)_ = 0.456, *p* = .713). Univariate analysis showed significant results for some variables in each of the three questionnaires for the differentiation of high vs. low levels of perceived social support, whereas the time of disclosure showed no significant influence on the outcome variables at all (see Table 2).

**Table 2:** **Univariate comparison of outcome variables between individuals with first disclosure in childhood and individuals with first disclosure in adulthood, using social support as covariate**

	Childhood disclosure mean (SE)	Adulthood disclosure mean (SE)	F_D_	*P*_D_	*part. Eta* ^*2*^	F_S_	*p*_*S*_	*part. Eta* ^*2*^
Status of Mental Health (T-Scores)								
Somatization^a^	62.39 (1.82)	64.51 (1.23)	0.916	.340	.006	6.144	.014	.037
Obsession- Compulsion^a^	62.41 (1.89)	61.32 (1.28)	0.229	.633	.001	2.682	.103	.017
Interpersonal Sensitivity	64.47 (1.71)	64.82 (1.15)	0.028	.867	.000	8.346	.004	.050
Depression^a^	65.37 (1.68)	66.85 (1.12)	0.534	.466	.003	3.695	.056	.023
Anxiety^b^	63.99 (1.93)	66.01 (1.29)	0.754	.386	.005	7.643	.006	.046
Hostility^b^	63.12 (1.75)	62.59 (1.17)	0.063	.802	.000	0.250	.617	.002
Phobic Anxiety^a^	65.39 (1.79)	65.18 (1.21)	0.009	.926	.000	7.471	.007	.045
Paranoid Ideation^a^	67.15 (1.40)	67.68 (0.94)	0.099	.754	.001	12.071	.001	.071
Psychoticism^b^	4.85 (1.76)	65.99 (1.17)	0.287	.593	.002	5.922	.016	.036
Global Severity Index^b^	67.76 (1.74)	69.40 (1.17)	0.607	.437	.004	8.482	.004	.051
PTSD symptoms								
Cluster B^a^	14.64 (0.78)	14.91 (0.52)	0.082	.775	.001	7.100	.009	.043
Cluster C^c^	17.25 (1.00)	18.06 (0.66)	0.455	.501	.003	6.686	.011	.041
Cluster D^a^	13.44 (0.76)	13.31 (0.51)	0.019	.889	.000	3.343	.069	.021
Total^c^	45.44 (2.33)	46.33 (1.55)	0.100	.752	.001	6.774	.010	.042
Intensions and emotions during disclosure								
Urge to talk^a^	8.65 (0.65)	8.66 (0.44)	0.000	.995	.000	0.486	.487	.003
Reluctance to talk^a^	8.96 (0.79)	8.77 (0.54)	0.040	842	.000	4.207	.042	.026
Emotional reactions during disclosure^a^	11.77 (0.83)	12.55 (0.56)	0.595	.442	.004	9.284	.003	.055

*Note*. ^a^N = 162. ^b^N = 161. ^c^N = 159. *P*_D_ Probability Disclosure, *P*_*S*_ Probability Social Support

Hostility was found to be one of the dominant social affects in our population, in 98 participants (60.1 %) the T-score of this subscale of BSI exceeded the cut-off of 63. Predictors for the severity of hostility were investigated. As covariates in the binary-logistic regression model questionnaire data of DLE, RPSSQ and PCL-C were used as well as the dichotomous variables current partnership status (yes = 98 (60.1 %)/no = 64 (39.3 %/1 MD) and sexual (yes = 119 (73.0 %)/no = 43 (26.4 %)/1 MD), physical (yes = 94 (57.7 %)/no = 68 (41.7 %)/1 MD) and emotional violence experiences (yes = 130 (79.8 %)/no = 32 (19.6 %)/1 MD) in childhood (in yes/no-format). The model fit was significant (Chi^2^ = 88.532, df = 9, *p* < .001) with a rate of explained variance of 58.8 % for the combination of the two predictors physical violence experienced in the past (Regression Coefficient = −1.130, *p* = .047, Odds Ratio = 0.323, CI (95 %) = 0.106 – 0.984) and severity of posttraumatic symptoms (Regression Coefficient = 0.146, *p* < .001, Odds Ratio = 1.157, CI (95 %) = 1.101 – 1.217) producing an overall rate of 128 out of 156 participants classified correct (82.1 %; see Table 3).

**Table 3:** **Binary logistic regression for predicting the severity of hostility using current disclosure, perceived social support, actual partnership (yes/no), type of violence experienced (yes/no), severity of posttraumatic symptoms**

Variables	Regression coefficient	*SE*	Wald		*p*	Exp(B)
Urge to talk	−0.074	0.060	1.548	1	0.214	0.928
Reluctance to talk	−0.100	0.054	3.387	1	0.066	0.095
Emotional reaction	0.000	0.055	0.000	1	1.944	1.000
Partnership (y/n)	0.221	0.489	0.205	1	0.651	1.248
Social support perceived	−0.036	0.031	1.340	1	0.247	0.965
Physical violence	1.130	0.568	3.953	1	0.047	0.323
Sexual violence	−0.362	0.623	0.338	1	0.561	0.696
Emotional violence	0.131	0.628	0.043	1	0.835	1.140
Severity of posttraumatic symptoms	0.146	0.026	32.738	1	<0.001	1.157
constant	−2.981	1.436	4.309	1	0.038	0.051

*Note.* Variable entered on step 1: urge to talk, reluctance to talk, emotional reaction, partnership, social support perceived, physical violence, sexual violence, emotional violence, severity of posttraumatic symptoms. *SE* = standard error, *df* = degrees of freedom

## Discussion

The results of this study are in line with previous findings on perceived social support on mental health (Kaniasty & Norris 2008) and PTSD (Brewin et al. 2000). Those with high levels of perceived social support have fewer emotional reactions when currently speaking about the past IA. Furthermore, the level of symptoms manifested in the group with a higher level of perceived social support is smaller, but not in all scales of psychopathology. The timing of disclosure did not reveal a relation with the current level of mental health, for both, the posttraumatic stress and comorbid symptoms. Additionally, we found some evidence that hostility is impacted by the experience of physical violence, and the severity of posttraumatic symptoms. Living with a partner does not show any correlation, as well as the reactions of disclosure and further forms of IA-related violence experiencing during the childhood.

Perceived social support, that is being embedded in social interactions that provide individuals with actual assistance perceived to be caring, and having the notion that support is available at any time, might buffer trauma related psychopathology, thus perceived social support might be an influential factor for the recovery. Direct effects of social support occur where health is improved or maintained, irrespective the stress levels. A perception that includes the idea that others are willing to help could result in an increased overall positive affect, a higher self-esteem, and more control over the environment (Cohen & Syme 1985). Direct effects of perceived social support suggest that a direct benefit could occur as a result of integrated membership in a social network (Cohen & Syme 1985), the latter was not given, since the social support sources differed within the sample. Our results corroborate research on perceived social support and PTSD in a specific sample of survivors of childhood abuse and maltreatment in institutions. The institutional background provided control over the entire life of those children. Caring social interactions were not inherent, but stemmed mostly form outside the system. Some researchers, (Sarason et al. 1994) conceptualized perceived social support as a manifestation of a relatively stable personality trait. This might be the case in our sample. However, looking into aspects of personality with respect to perceived social support would need a longitudinal design, which was not given in our study. A clear distinction sustained competencies to mobilise social support and social influences for future research is needed. However, it remains unclear which model of perceived social support is the most relevant for a better understanding of our results. Most researchers looking into the relation of social support and PTSD use the stress buffering model, to explain the symptom reduction resulting from higher social support. More research with a clearer concept of the effects of social support would be needed.

It is noteworthy that the timing of disclosure in itself does not indicate any significant effect on mental health, neither on PTSD symptoms nor the intensity of emotions while addressing the abuse. Opportunities for the Austrian survivors of IA within the Catholic Church to make timely disclosures following their experiences were rare. Reasons for this might have been witnessing a peer’s unsuccessful attempt to confide in someone, deciding to forgo the disclosure when confronted with disbelief when sharing the experiences with peers, the fear of some form of betrayal (Freyd 1996), or attempting to forget by not talking about the experience at all. Pennebaker (Pennebaker 1997) addressed a special aspect of this issue with the term ‘silent disclosure’. He postulated that writing down these experiences would help to cope with the related feelings and thoughts, especially in the case of betrayal trauma. But it would prevent the social environment from listening, and from negative reactions towards the victim. Social affects, related to the betrayal aspect and to the dissociative features that characterize disclosure might shape memories related to late disclosure and negatively impact the symptoms (Maercker & Horn 2013). The silent disclosure might explain that disclosure at any time after the experience does not show an impact on the level of mental health. From interviews with the participants of our study we have learnt that quite a number of them have written down their past experiences, but kept them secret from the public. Only recently, some survivors published autobiographies (Pirker 2012).

However, for the timing of disclosure inconsistent definitions can be found (O’Leary et al. 2010; Ruggiero et al. 2004). We used a combination of time between first exposure to abuse and first disclosure (which for all participants was within the range of three years) and the definition of childhood vs. adulthood disclosure (all of the participants of the early disclosure group first reported about the abuse within an age of 18 years), as we consider the differentiation between childhood and adulthood as the main criterion. Our results could not contribute to better understand the aspect of timing for disclosure which might be related with the distinction of times for disclosure.

Another aspect that is related to the amount and the quality of social support is one’s own attitude towards close others. Social affects shape the perception and the interaction. Hostility which was predicted by physical violence and the severity of the PTSD symptoms filters the perception of social support negatively and might reduce the concrete amount of support perceived (Kotler et al. 2001). However, there revealed some evidence for the interactions postulated, but further research is needed to provide detailed evidence for the interactions that explain the mutuality between the situation of an individual and the posttraumatic outcome.

### Limitations

The problem with all of the available research on disclosure is the lack of a control group. We compared those who made early disclosure to those who made late disclosures, but we lack information on those who make no disclosure. The non-disclosure group would have been the best control sample, but they remain in the shadows. An additional limitation is the fact that we had been researching survivors in the recall condition on average 45 years after exposure. In their sample of survivors of political suppression, Müller *et al.* (Müller et al. 2000) consider a recall condition of 25 years as possibly too long to research memories about disclosure attitudes and reactions. Not addressing disclosure in a research project which focuses on survivors who disclose abuse to a commission seems to be even less appropriate than asking for a recall dating back 45 years. We shared the dilemma of how to treat the topic of disclosure with the survivors, concluding that each survivor has to decide whether he or she will make a disclosure, while the research team has to decide whether to ask for disclosure. Both function in a recall-condition that might result in a shaped reality, according to Edwards, Holden, Felitti, and Anda (Edwards et al. 2003). While our findings might reflect a deficit in terms of underreporting, they do not reflect inflated symptoms. A further limitation is given by the rather small rate of respondents which is in accordance with other studies with victims of IA within the Catholic Church (O’Leary et al. 2010; Flanagan-Howard et al. 2009). This response rate might result from an overall shyness to disclose the experienced IA, but also from the characteristics of the sample which is dominated by male (Dorahy & Clearwater 2012).

## Conclusion

Our results provide some insight into the role of disclosure and social support in a sample of long-term survivors from institutional child abuse. Highly perceived levels of social support seem to play a crucial role in current mental health, but this hypothesis is weakened by a possible interference of a lasting competence to receive social support versus social influences. Future research should thus disentangle perceived social support into a sustained competence to mobilise lasting social support versus socially influenced factors to provide more clarity about the positive association, *e.g.*, by integrating questionnaires looking for support seeking behaviour. The aspect of the timing of disclosure itself seemed to be less relevant for long-term survivors. Future research on disclosure should address this point by developing adequate models of disclosure. For clinical purposes the factor hostility might become meaningful to address as hostility might impact the needed trust for the treatment process. Skills to better regulate negative emotions are crucial for stabilization (Stevens et al. 2013).
